# Cytokeratin 19 promoter directs the expression of Cre recombinase in various epithelia of transgenic mice

**DOI:** 10.18632/oncotarget.15435

**Published:** 2017-02-17

**Authors:** Gui-Feng Zhao, Shuang Zhao, Jia-Jie Liu, Ji-Cheng Wu, Hao-Yu He, Xiao-Qing Ding, Xue-Wen Yu, Ke-Qiang Huang, Zhi-Jie Li, Hua-Chuan Zheng

**Affiliations:** ^1^ Department of Experimental Oncology and Animal Center, Shengjing Hospital of China Medical University, Shenyang 110004, China; ^2^ Office of Administration, Jinzhou Medical University, Jinzhou 121001, China

**Keywords:** Cre recombinase, transgenic mouse, cytokeratin 19, PTEN, carcinogenesis

## Abstract

Cytokeratin 19 (K19) is expressed in various differentiated cells, including gastric, intestinal and bronchial epithelial cells, and liver duct cells. Here, we generated a transgenic mouse line, K19-Cre, in which the expression of Cre recombinase was controlled by the promoter of K19. To test the tissue distribution and excision activity of Cre recombinase, K19-Cre transgenic mice were bred with Rosa26 reporter strain and a mouse strain that carries PTEN conditional alleles (PTEN^Loxp/Loxp^). At mRNA level, Cre was strongly expressed in the stomach, lung and intestine, while in stomach, lung, and liver at protein level. The immunoreactivity to Cre was strongly observed the cytoplasm of gastric, bronchial and intestinal epithelial cells. Cre activity was detectable in gastric, bronchial and intestinal epithelial cells, according to LacZ staining. In K19-Cre/PTEN ^Loxp/Loxp^ mice, PTEN was abrogated in stomach, intestine, lung, liver and breast, the former two of which were verified by *in situ* PCR. There appeared breast cancer with PTEN loss. These data suggest that K19 promoter may be a useful tool to study the pathophysiological functions of cytokeratin 19-positive cells, especially gastrointestinal epithelial cells. Cell specificity of neoplasia is not completely attributable to the cell-specific expression of oncogenes and cell-specific loss of tumor suppressor genes.

## INTRODUCTION

The mouse stomach consists of squamous forestomach, glandular corpus and antrum. The gastric unit in the corpus region has pit cells, parietal cells, chief cells, mucous neck cells, stem cells, and enteroendocrine cells, whereas there are only mucus-producing pit and neck cells in gastric units of distal antrum. The mucus-producing pit cells are localized to the edge of the gastric mucosa. Parietal cells in oxyntic and cardiac glands secrete HCl by hydrogen potassium ATPase. Chief cells in oxyntic gland release pepsinogen, chymosin, lipase enzymes and leptin. The three above-mentioned cells are believed to differentiate from stem cells of gastric isthmus and their dysfunction has been reported to be involved in atrophic gastritis and gastric cancers [1–2].

Gene targeting is a powerful technique to investigate the functions of specific gene *in vivo* because the general inactivation of the target genes might lead to early embryonic lethality. Cre-Loxp system provides a powerful approach to enable a cell- or tissue- specific deletion of a target gene. In a previous work, the promoters of the calpain-8 (Capn8) [1], β-subunit of H1-, K1-ATPase (Atp4b) [2], and villin [3] have been used to drive Cre gene expression in pit cells, parietal cells, gastric isthmus cells, and gastric progenitors, respectively. Although Means et al. [4] established a K19-CreERT mouse by tamoxifen activation using cytokeratin 19 (K19) promoter, it is inconvenient to establish the spontaneous cancer model because of tamoxifen administration. In K19-CreERT mouse, leaky Cre activity could be detected in less than 1% of gastric and intestinal epithelial cells in the absence of tamoxifen, but tamoxifen treatment in postnatal animals induced widespread DNA recombination in epithelial cells of pancreatic ducts, hepatic ducts, stomach, and intestine.

Cytokeratins are intermediate filaments for the maintenance of the cytoskeleton and classified into types I (cytokeratin 9-20) and II (cytokeratin 1-8). Cytokeratin 19 is expressed in multiple cell types from the epiblast stage and is maintained in multiple epithelial cell types of later embryonic and postnatal stages, including the pancreatic ducts and liver duct cells [4, 5]. Oshima et al. [6] build up K19-Wnt1/C2mE mice of gastric cancer because K19 promoter directed specific expression of Wnt1, Cox-2 and PGE in the subpopulation of gastric progenitors. To study the role of targeted ablation of some genes in gastric carcinogenesis, we generated the K19-Cre mouse and observed its conditional knockout of PTEN in gastric progenitors.

## RESULTS

**Figure 1:**
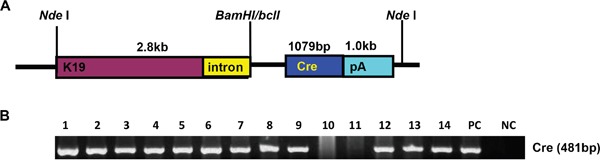
The establishment and genetic screening of K19-Cre transgenic mice The transgenic mouse of Cre was established according to schematic diagram using K19 promoter **A**. We found 12 founders with Cre positive by PCR of mouse tail DNA **B**. Note: PC, positive control, pBS185 plasmid containing Cre gene; NC, negative control, no DNA template.

**Figure 2:**
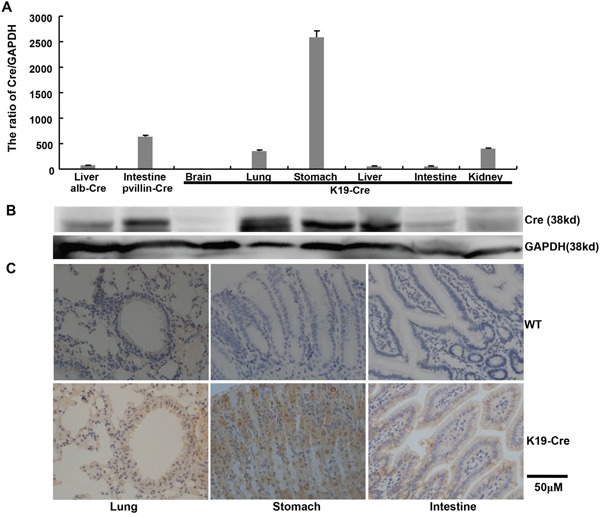
Cre expression in the organs of K19-Cre transgenic mice Cre expression levels were detected by real-time RT-PCR **A**., Western blot **B**. and immunohistochemistry **C**. with the liver of alb-Cre mouse and intestine of pvillin-Cre mouse as positive control. GAPDH was employed as an internal control in the experiments of RT-PCR and Western blot. WT, wild-type C57 mouse.

We generated a transgenic mouse strain (K19-Cre) in which Cre recombinase expression was under the control of a 2.8-kb promoter of the mouse cytokeratin 19 gene (K19). A Cre-coding region was inserted between K19 promoter and SV40 poly A as indicated in Figure 1A. The 5.6-kb linearized fragment was isolated using *Nde*I and microinjected into the pronuclei of fertilized oocytes for the generation of the transgenic mice. Mouse tail DNA was subjected to PCR targeting Cre and clear bands were considered as positive (Figure 1B). At mRNA level, Cre was strongly expressed in the stomach, lung and intestine. Western blot showed Cre overexpression in stomach, lung, and liver (Figure 2A and 2B).

**Figure 3:**
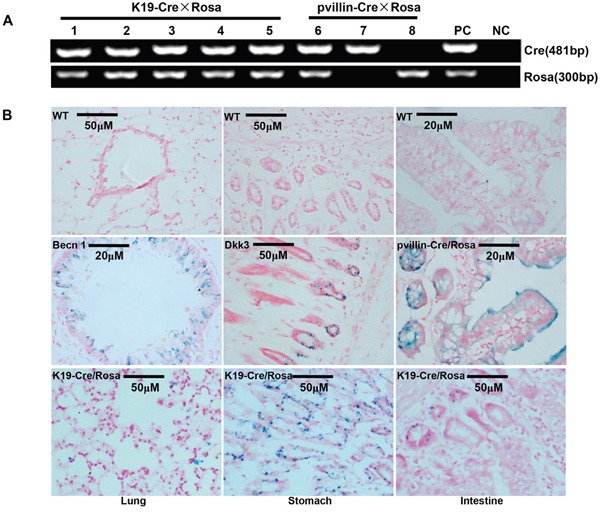
Cre activity in various tissues of K19-Cre transgeneic mice After mated with Rosa26-lacZ mouse **A**., Cre activity in K19-Cre/Rosa transgenic mice was visualized in lung, stomach and intestine by lacZ staining **B**. Wild-type C57 mouse (WT) was employed as a negative control and the target knockout mice of B6.Cg-*Dkk 3*^tm1^, B6.Cg-*Becn1* ^tm1^ pvillin-Cre/Rosa as a positive control due to the existence of endogenous LaZ gene. Note: PC, positive control, tail DNA of pvillin- Cre mouse or Rosa26-lacZ; NC, negative control, no DNA template.

The immunoreactivity to Cre was strongly observed in the cytoplasm of gastric, bronchial and intestinal epithelial cells (Figure 2C). To identify the exact cell types in which Cre recombinase performs its excision function, we bred K19-Cre transgenic mouse with the reporter mouse Rosa26-LacZ to activate β-galactosidase (Figure 3A). As shown in Figure 3B, galactosidase was detectable in gastric, bronchial and intestinal epithelial cells using x-gal as subtract. Wild-type C57 mouse (WT) was employed as a negative control and the target knockout mice of B6.Cg-*Dkk 3*^tm1^, B6.Cg-*Becn1* ^tm1^, pvillin-Cre/Rosa as positive control due to the existence of endogenous LaZ gene respectively.

**Figure 4:**
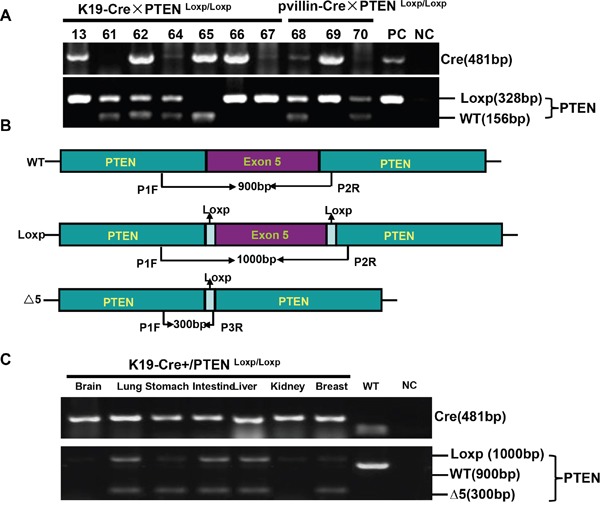
K19-Cre-mediated PTEN deletion in conditional knockout mice After mated K19-Cre mice with B6.129S4-PTEN^tm1^ mice, the founders were verified with tail DNA by PCR **A**. Primers were designed targeting PTEN gene to differentiate the deletion of exon 5 **B**. The different tissues from K19-Cre/PTEN ^Loxp/Loxp^ mice were subjected to DNA extract and PCR amplification using above-mentioned primers **C**. Note: PC, positive control, the tail DNA of pvillin-cre mouse or wild-type C57 mouse in figure A; NC, negative control, no DNA template; WT, wild-type C57 mouse.

**Figure 5:**
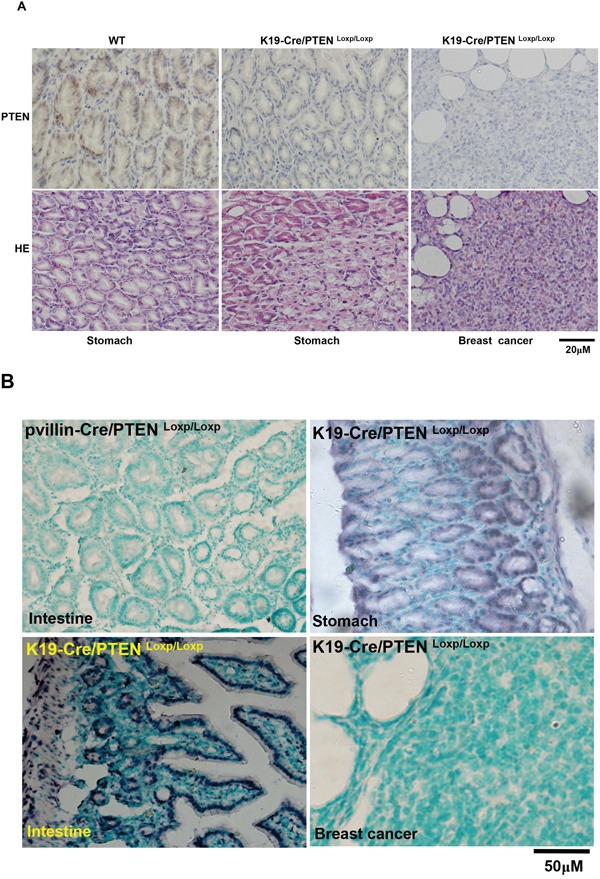
Breast carcinogenesis in transgenic mice with tissue-specific abrogation of PTEN There appeared the expression loss of PTEN in gastric mucosa and breast cancer in K19-Cre /PTEN ^Loxp/Loxp^ mice by immunohistochemistry **A**. *In situ* PCR showed that the deletion of PTEN exon 5 was deleted in breast cancer, gastric and intestinal epithelium with the intestine of pvillin-Cre/PTEN^Loxp/Loxp^ as a positive control **B**. WT, wild-type C57 mouse; HE, hematoxylin-eosin staining.

To check the excision activity of Cre recombinase, K19-Cre transgenic mice were crossed with the mice carrying PTEN conditional alleles (PTEN^Loxp/Loxp^), in which both Loxp sites are inserted into introns 4 and 5 of PTEN. Therefore, Cre can delete the exon 5 to inactivate PTEN gene (Figure 4A). Additionally, we also designed primers around exon 5 as shown in Figure 4B and found that exon-abrogated and small-size bands in lung, stomach, intestine, liver and breast of K19-Cre/PTEN ^Loxp/Loxp^ mice (Figure 4C). In the target knockout mice, PTEN was not expressed in gastric mucosa and breast cancer according to the results of immunohistochemistry (Figure 5A). In addition, we found no PTEN signal in some gastric and intestinal epithelial cells according to the data of *in situ* PCR targeting only exon 5 (Figure 5B). It was noted that breast cancer was found with no PTEN signal in both DNA and protein levels (Figure 5A-5B). The intestine of pvillin-Cre/PTEN ^Loxp/Loxp^ mouse showed no DNA signal of PTEN exon 5, and consequently was used as a positive control.

## DISCUSSION

According to the mucins’ expression, Lauren [7] believed that the intestinal-type gastric carcinoma originates from the regenerating epithelium in chronic atrophic gastritis with incomplete type of intestinal metaplasia, while diffuse-type carcinoma from non-metaplastic gastric epithelium. However, it is well known that gastric signet ring cell carcinoma originates from globoid dysplasia (also called as *in situ* signet ring cell carcinoma), which developed from intestinal metaplasia [8, 9]. Adenomatous, regenerative and cryptal dysplasia might be aggravated into intestinal-type carcinoma [10]. Zheng et al. [11] found that the difference in pathobiological features between intestinal and diffuse components of mixed-type (MT) carcinoma was smaller than that between pure intestinal- and diffuse-type ones, indicating that different components of MT carcinoma might originate from common stem cells [12], but follow distinct histogenic pathways. However, no evidences are provided about what cells play an important role in gastric histogenesis and what their responsible gastric cancers are characterized by.

Until now, Syder et al. [13] established a transgenic mouse model of a SV40 T antigen, which was activated by Atp4b-Cre mice in acid-producing parietal cells. Metastatic gastric cancer was found and closely linked to transdifferentiation of parietal cell progenitors to a neuroendocrine phenotype. Shimada et al. [14] demonstrated that double conditional knockout of CDH1 and p53 caused diffuse-type gastric carcinoma using Atp4b-Cre mice. In transgenic mice overxpressing TGFα, parietal and chief cells were specifically depleted from the glandular mucosa, and zymogen granule-containing cells in the parotid gland underwent redifferentiation to form tubular complexes, and collections of ductularlike structures, which are frequently observed in gastric cancer [15, 16]. Ras activation in chief cells of Mist1-Kras mice led to the full range of metaplastic lineage transitions, including spasmolytic polypeptide- expressing metaplasia and intestinal metaplasia, which might be reversed by suppressing Ras signaling by MEK inhibition [17]. Clinical and experimental evidences indicate that the dysfunction and loss of chief and parietal cells are closely linked to gastric carcinogenesis [13–20].

Cytokeratin 19 promoter guides the overexpression of oncogenes to result in gastric neoplasia, while pulmonary neoplasia was found in transgenic mice of K19-JCV T antigen [6, 21, 22]. Therefore, it is hypothesized that genetic alteration in cytokeratin-19-positive cells contributes to carcinogenesis with cell specificity. To explore the effects of tumor suppressor genes in carcinogenesis, we successfully established a transgenic animal model of K19-Cre, in which Cre was strongly expressed in gastric, bronchial and intestinal epithelial cells. High Cre content in liver is due to its overexpression in hepatic bile ducts [4]. To determine Cre activity, we employed Rosa26 reporter gene mice containing a floxed neomycinstop cassette of LacZ. Once the activation of Cre, the stop cassette is removed, and LacZ is activated by the Rosa promoter. In line with the opinion that K19-CreERT allows inducible recombination in bile duct and gastrointestinal epithelial cells, we also found the same results as Cre expression, suggesting that K19-Cre mice might be employed to delete Loxp-flanked DNA fragment for conditional knockout.

PTEN protein acts as a phosphatase to dephosphorylate PIP3 for the inhibition of the Akt signaling pathway. PTEN's phosphatase activity may cause cell arrest. Frequent genetic inactivation of PTEN occurs in glioblastoma, endometrial and prostate cancers, and reduced expression is found in many other tumor types such as lung, colorectal, gastric and breast cancers. However, its cell-specific knockout PTEN only causes hepatocellular cancer, urothelial carcinoma of bladder, ureter and kidney, squamous cell carcinoma of vagina and rectum, colonic adenocarcinoma, prostate cancer, papillomas, squamous cell carcinomas and T-cell lymphoma [23]. Shore et al. [24] found that luminal-specific PTEN loss caused increased proliferation of hormone receptor-negative cells and the decreased percentage of hormone receptor-positive cells with misoriented cell divisions and mislocalization of cells to the intraluminal space of mammary ducts. However, target deletion of PTEN leads to precocious development and neoplasia in the mammary gland using MMTV-Cre transgenic mice [25] and there appeared breast cancer in ErbB2/Neu-overexpressing/PTEN-deficient mice [26].

Therefore, we bred K19-Cre/PTEN^Loxp/Loxp^ and found that PTEN loss was observed in the tissues of lung, stomach, intestine, liver and breast. Interestingly, breast cancer was found in K19-Cre-mediated conditional knockout mice of PTEN. In combination of these findings, we speculated that there was some leakage of K19 promoter, even to breast. Furthermore, cell specificity of neoplasia is not completely attributable to the cell-specific expression of oncogenes and cell-specific loss of tumor suppressor genes. Cell microenviroments also play an important role in this event, including metabolism, chemical modification of target protein, compensation of down-regulated proteins and their partners. To confirm the PTEN DNA, we designed the primers targeting exon 5 to differentiate the wild-type and mutant PTEN gene by *in situ* PCR, which differentiated the PTEN deletion in gastric and intestinal epithelial cells.

In conclusion, we established a K19-Cre transgenic mouse line, which is able to drive Cre transgene expression in gastric, bronchial, intestinal and breast epithelial cells. The establishment of K19-Cre transgenic mouse line will provide a valuable tool for studying the genetic mechanisms underlying the physiological functions of cytokeratin 19-positive cells, especially gastrointestinal epithelial cells.

## MATERIALS AND METHODS

### Transgene construction and genotyping

To insert starting code between Kozak and nuclear localization sequences, Cre gene (1079bp) was amplified using primer set (forward, 5'-TGATCAACCATGGATGCCACCAAAGAAGAAGAGAAAGG-3' and reverse, 5'-TGATCACTAATCGCCATCTTCCAGCAGGC GCA CC-3') and the template DNA of pBS185 (Addgene, USA). The phosphorylated products were ligated into *Hinc*II-digested and dephosphorylated pBluescript K (+). The *Bcl*I-digested Cre fragment was compatibly ligated with *Bam*I-digested K19-Cox-2 (Kindly presented by Prof. Oshima, Kanazawa University, Japan). The 4.8-kb linearized insert was excised from the vector backbone by *Nde*I digestion and purified using QIAquick Gel Extraction Kit (QIAGEN, Chatsworth, CA). The inserts were microinjected into the pronuclei of fertilized FVB mouse oocytes to generate the transgenic mice.

### Animal care

K19-Cre, wild-type C57, B6.129S4-PTEN ^tm1^Hwu/J, pvillin-Cre and alb-Cre mice (Jackson Lab), Rosa26-lacZ reporter mice (Kindly presented by Prof. Zhi-hong Zheng, China Medical University), B6.Cg-*Dkk3* ^t^ ^m1^ (Kindly presented by Prof. Hiromi Kumon, Okayama University), and B6.Cg-*Becn1* ^tm1^(Purchased from UCDAVIS KOMP Repository) mice were maintained in specific-pathogen-free room. We crossed K19-Cre and pvillin-Cre with Rosa26-lacZ reporter mice or B6.129S4-PTEN ^tm1^Hwu/J to obtain the K19-Cre/Rosa, K19-Cre/PTEN ^Loxp/Loxp^, pvillin-Cre/Rosa and pvillin-Cre/PTEN ^Loxp/Loxp^. Every four mice were housed to a plastic cage with paper chips for bedding. All had access to standard rodent food (Beijing HFK Bioscience) and water, and were housed in a temperature-controlled animal room with a 12-h light/dark illumination cycle. Animal use procedures were in accordance with the Guide for the Care and Use of Laboratory Animals and approved by the Committee on Animal Experimentation of our hospital.

### PCR

DNA was extracted from the mouse tail, brain, lung, stomach, intestine, liver, kidney and breast by proteinase K digestion and phenol/chloroform. To confirm the genetic phenotype, we performed PCR using tail genomic DNA as template and targeting Cre, Rosa and PTEN with Hotstart polymerase (Takara, Japan). The primers for Cre were forward, 5'-GCCTGCATT ACCGGTCGATGC-3' and reverse, 5'-CAGGGTGTTATAAGCAATCCC-3' (481bp). The primers for Rosa were forward: 5'-AAAGTCGCTCTGAGTTGTTAT-3' and reverse: 5'-GCGA AGAGTTTGTCCTCAACC-3' (300bp). The primers for PTEN were forward: 5'-CAAGCAC TCTGCGAACTGAG-3' and reverse: 5'-AAGTTTTTGAAGGCAAGATGC-3' (Δ5, 328bp and WT, 156bp). To verify the conditional knockout of PTEN in specific organ, we performed PCR using organ genomic DNA targeting PTEN with Hotstart polymerase. The primers were designed as previously reported [27]: P1 forward, 5'-ACTCAAGGCAGGGATG AGC-3', P2 reverse, 5'-AATCTAGGGCCTCTTGTGCC-3' and P3 reverse, 5'-GCTTGATATCGAATTCC TGCAGC-3' (Figure 4B).

### Real-time RT-PCR

Total RNA was isolated from gastric mucosa, liver, intestinal mucosa, brain, lung and kidney. RNA was subjected to cDNA synthesis using avian myeloblastosis virus transcriptase and random primer (Takara, Japan). PCR was carried out using SYBR Premix Ex Taq™ II kit (TaKaRa, Japan). Cre gene was described as mentioned above Glyceraldehyde-3-phosphate dehydrogenase (GAPDH) primers were forward, 5'-CAACGACCCCTTCATTGAC C-3' and reverse, 5'-GGCTTCCCGT TGATGACAAG-3' were used as an internal control. The expression level of Cre was expressed as 2^-ΔCt^, where ΔCt = Ct (Cre) – Ct (GAPDH). Additionally, the minimum expression level was considered as “1”.

### Western blot

The protein was extracted by homogenizing in RIPA lysis buffer and subjected to concentration assay. Denatured proteins were resolved on a SDS polyacrylamide gel and then transferred to PVDF membranes. After blocking with 5% skim milk, they were incubated with antibody against Cre (Novus, USA) or GADPH (Sigma, USA), and then HRP- conjugated secondary antibody (DAKO). Finally, coloring is performed using ECL detection solution (Santa Cruz) in Image Quant LAS4010 (GE Bioscinece).

### LacZ staining

The tissues of the double transgenic mice and some conditional knockout mice were fixed in 4% paraformaldehyde at room temperature. Then they were fixed in 30% (w/v) sucrose solutions (dissolved to 0.1M phosphate buffer) at 4°C for 24h. Subsequently, tissues were embedded into OCT compound, and sectioned at 4μm. Sections were then dipped in 0.1M phosphate buffer containing 2 mmol/L MgCl_2_, 0.2% NP-40, and 0.1% Na-deoxycholate. The staining was carried out in the washing buffer supplemented with 1 mg/mL x-gal, 6M potassium ferrocyanide, and 6M potassium ferricyanide for 48 h. The sections were subsequently washed with 0.1M phosphate buffer and counterstained with nuclear fast red.

### Immunohistochemistry

The formalin-fixed and paraffin-embedded block was cut into 4μm-thick sections for hematoxylin-eosin staining. The sections were also deparaffinized, dehydrated, and subjected to antigen retrieval using target retrieval solution (TRS, DAKO, USA) as Kumada et al. [28] described. Three percent hydrogen peroxide was used to block endogenous peroxidase activity, and five percent bovine serum albumin to prevent non-specific binding. The sections were incubated with rabbit anti-Cre (Novus) or anti-PTEN (Cell Signaling) antibody for 60 min, then treated with the anti-rabbit Envison-PO (DAKO) antibody for 60 min. All slides were developed for coloring with 3, 3'-diaminobenzidine and counterstained with Mayer's hematoxylin. Omission of the primary antibody was used as a negative control.

### *In situ* PCR

Ten-μm-thick sections were deparaffinized and subjected to the digestion of 20μg/ml proteinase K for 15 min at 37°C. After rinsing with PBS, the sections were fixed with 4% neutral paraformadehyde and washed with 2×SSC. Then 125μl of RT-PCR solution (0.2μM primers of PTEN targeting exon 5, 0.125 nM DIG-11-dUTP, 2.5 mM MgCl_2_, 1×PCR buffer, 6.25 U Taq polymerase) was placed into a membrane tissue sealing and PCR was carried out as follows: 42 °C for 5 min and 94 °C for 20 s, followed by 20 cycles of 92 °C for 15 s, 55 °C for 15 s and 72°C for 30 s, finally 72°C for 5 min. The primers for PTEN were forward, 5'-ACCATAACCCACCACAGC-3' and reverse, 5'-TTACACCAGTCCGTCCCT-3' (156bp). After that, we washed the slides with 2×SSC and incubated the tissue with blocking solution (100μg/ml Salmon testis DNA, 100μg/ml yeast tRNA, and 5% BSA in PBS). The sections were washed using TBST and incubated with anti-digoxigenin antibody conjugated with alkaline phosphatase. The slides were then washed in 5 min and immersed in solution II (100 mM Tris-HCl pH 9.5, 100 mM NaCl and 50 mM MgCl_2_) and incubated with an anti-digoxigenin antibody coupled to AP overnight and followed by NBT/BCIP as chromogen. Finally, counterstaining of the tissue was performed with methyl green.
